# Yoga compared to non-exercise or physical therapy exercise on pain, disability, and quality of life for patients with chronic low back pain: A systematic review and meta-analysis of randomized controlled trials

**DOI:** 10.1371/journal.pone.0238544

**Published:** 2020-09-01

**Authors:** Feilong Zhu, Ming Zhang, Dan Wang, Qianqin Hong, Cheng Zeng, Wei Chen

**Affiliations:** 1 The Affiliated Xuzhou Rehabilitation Hospital of Xuzhou Medical University, Xuzhou Rehabilitation Hospital, Xuzhou, China; 2 Department of Rehabilitation Medicine, XuZhou Central Hospital, Xuzhou, China; 3 Department of Rehabilitation Medicine, The First Hospital of Putian City, Putian, China; Universita degli Studi di Ferrara, ITALY

## Abstract

**Background:**

Chronic low back pain (CLBP) is a common and often disabling musculoskeletal condition. Yoga has been proven to be an effective therapy for chronic low back pain. However, there are still controversies about the effects of yoga at different follow-up periods and compared with other physical therapy exercises.

**Objective:**

To critically compare the effects of yoga for patients with chronic low back pain on pain, disability, quality of life with non-exercise (e.g. usual care, education), physical therapy exercise.

**Methods:**

This study was registered in PROSPERO, and the registration number was CRD42020159865. Randomized controlled trials (RCTs) of online databases included PubMed, Web of Science, Cochrane Central Register of Controlled Trials, Embase which evaluated effects of yoga for patients with chronic low back pain on pain, disability, and quality of life were searched from inception time to November 1, 2019. Studies were eligible if they assessed at least one important outcome, namely pain, back-specific disability, quality of life. The Cochrane risk of bias tool was used to assess the methodological quality of included randomized controlled trials. The continuous outcomes were analyzed by calculating the mean difference (MD) or standardized mean difference (SMD) with 95% confidence intervals (CI) according to whether combining outcomes measured on different scales or not.

**Results:**

A total of 18 randomized controlled trials were included in this meta-analysis. Yoga could significantly reduce pain at 4 to 8 weeks (MD = -0.83, 95% CI = -1.19 to -0.48, p<0.00001, I^2^ = 0%), 3 months (MD = -0.43, 95% CI = -0.64 to -0.23, p<0.0001, I^2^ = 0%), 6 to 7 months (MD = -0.56, 95% CI = -1.02 to -0.11, p = 0.02, I^2^ = 50%), and was not significant in 12 months (MD = -0.52, 95% CI = -1.64 to 0.59, p = 0.36, I^2^ = 87%) compared with non-exercise. Yoga was better than non-exercise on disability at 4 to 8 weeks (SMD = -0.30, 95% CI = -0.51 to -0.10, p = 0.003, I^2^ = 0%), 3 months (SMD = -0.31, 95% CI = -0.45 to -0.18, p<0.00001, I^2^ = 30%), 6 months (SMD = -0.38, 95% CI = -0.53 to -0.23, p<0.00001, I^2^ = 0%), 12 months (SMD = -0.33, 95% CI = -0.54 to -0.12, p = 0.002, I^2^ = 9%). There was no significant difference on pain, disability compared with physical therapy exercise group. Furthermore, it suggested that there was a non-significant difference on physical and mental quality of life between yoga and any other interventions.

**Conclusion:**

This meta-analysis provided evidence from very low to moderate investigating the effectiveness of yoga for chronic low back pain patients at different time points. Yoga might decrease pain from short term to intermediate term and improve functional disability status from short term to long term compared with non-exercise (e.g. usual care, education). Yoga had the same effect on pain and disability as any other exercise or physical therapy. Yoga might not improve the physical and mental quality of life based on the result of a merging.

## Introduction

Chronic low back pain (CLBP) refers to the low back, lumbosacral and sacroiliac pain over 3 months which is sometimes accompanied by radiation pain in the lower extremities, and non-specific low back pain is pain not attributed to a recognizable pathology [1]. Chronic low back pain often causes pain and functional disability [2], which has a negative effect on the personal quality of life [3]. 7.3% of the world's population including approximately 10% adults in the United States [4] are suffering from pain obsession and limited function caused by low back pain [5]. Nevertheless, generally speaking, many patients are not satisfied with the treatment of CLBP [6], and it is troublesome [7] for orthopedists, physical therapists, and patients.

The conventional treatments for low back pain are usual care and physical therapy exercises. Yoga is a mind-body exercise sometimes used for non-specific low back pain [8]. For professionals and patients dealing with CLBP, there is a need to know whether effective yoga practices compared with other treatments like standard medical care, exercises, traditional physical therapy. Unfortunately, previous studies have provided low-level evidence concerning the effects of yoga on chronic low back pain patients compared with non-exercise (e.g. usual care, education, a waiting list) [9]. In Nambi's [10] and Tekur's [11] original research, these results suggested that yoga provided better improvement in pain reduction and improvement in the quality of life in chronic back pain patients than general exercise programs. However, some other authors concluded that yoga provided similar improvement compared with physical therapy exercise in patients with CLBP [4, 12, 13]. In Cramer's [14] and Holtzman's [15] review of yoga for low back pain, they were uncertain whether yoga offered any advantages over traditional exercise programs. In Wieland's study, it was uncertain whether there was any difference between yoga and other exercises for back-related function or pain, and the study provided limited evidence on measures of quality life due to lacking studies for calculating effect sizes [8]. Thus, it is necessary to seek more relevant high-quality studies to provide additional information on comparisons between yoga and other physical therapy exercises for chronic low back pain [8]. Therefore, this review and meta-analysis aimed to systematically assess the effectiveness of yoga compared with non-exercise and physical therapy exercise on pain, disability, quality of life at different follow-up periods in patients with chronic low back pain.

## Methods

This study was registered in PROSPERO, and the registration number was CRD42020159865. The laboratory protocol was deposited in protocols.io, http://dx.doi.org/10.17504/protocols.io.bi6gkhbw. The Cochrane Handbook for Systematic Reviews of Interventions was strictly followed by authors to accomplish this project.

### Data sources

The PubMed, Embase, Web of Science, Cochrane Central Register of Controlled Trials databases were searched from their inception time to November 1, 2019. The language of publication was not restricted. In addition to the search of the electronic databases, we also searched Google and Google Scholar for Internet search, reference lists of included studies and relevant reviews by manual retrieval method. The full PubMed search strategy was detailed in the online S1 File.

### Inclusion and study selection

The studies needed to meet the following inclusion criteria to be included in the meta-analysis and review: clinical randomized controlled trials (RCTs); non-specific low back pain lasting for at least three months; participants age more than 18 years old without gender limitation; the experimental group took yoga intervention or in combination with other treatments regardless of the style of yoga, frequency, and duration of intervention; the control group received waiting-list, no treatment, a minimal intervention (e.g. education, booklets), usual care, or other active treatments, such as physical therapy, conventional therapeutic exercises; studies evaluating the effects of yoga on pain, disability, quality of life in individuals with chronic low back pain; studies providing sufficient data for calculating effect sizes. The exclusion criteria were as follows: studies were non-randomized controlled trials; wrong population, not related outcomes, no data reported for analysis, etc.

### Data extraction and synthesis

Data were extracted by two reviewers independently, and disagreements were discussed between reviewers to reach a consensus. A third reviewer made the final decision if disagreements were persisted. Detailed data were extracted which including the first author, publication year, country of publication, clinical status, number of participants, participant characteristics, experimental and control interventions, intervals of intervention, outcome measures, results, and adverse events. Missing data were handled by contacting the author with emails.

Firstly, two reviewers independently read the titles and abstracts of the searched studies to exclude irrelevant studies. Then, the full text of the remaining studies was to be read and make the final selection. The inter-rater agreement for selecting studies with the Kappa score was calculated by IBM SPSS Statistics 25. We classified waiting-list, no treatment, a minimal intervention (e.g. education, booklets), usual care as non-exercise. We utilized the baseline versus follow-up change scores comparison between groups for calculating effect sizes. Where no standard deviations (SDs) were available, they were calculated from standard errors (SEs), CIs, t or p values, or attempts were made to obtain the missing data from the authors by email [17]. If the author did not report the data in the paper but provided a graph with the data, we used GetData Digitizer version 2.20 software to extract data we need from graphs. In cases where both unadjusted and adjusted data were available in the paper, we used the adjusted data for our study. Review Manager 5.3 software and Stata 16.0 software were used in this article for data analysis. If units of measurement of this outcome were consistent, the mean difference (MD) and 95% confidence intervals (CI) were used for pooling effects. When combining this outcome measured on different scales, the standardized mean difference (SMD) with 95% confidence intervals (CI) were used. Meanwhile, subgroup analysis based on scales with the mean difference and 95% confidence intervals were conducted [18]. Statistical heterogeneity among the studies was assessed by the chi-square test and I^2^ value. The synthesis of included trials was considered as a significant heterogeneity if P < 0.1 or I^2^ > 0.5, and a random-effect model was carried out [19]. Furthermore, sensitivity analysis by excluding trials one by one or visualizing from the funnel plot, and subgroup analysis were conducted to explore the source of heterogeneity. Eventually, the funnel plot was carried out to assess the publication bias with Stata 16.0 software.

### Data analysis

We defined that outcomes were assessed at short-term (closest to six weeks), short to intermediate-term (closest to three months), intermediate-term (closest to six months), and long-term (closest to one year) follow up periods. Studies were analyzed separately for short-term, short to intermediate-term, intermediate-term, and long-term time points.

### Risk of bias assessment

The risk of bias was independently assessed by two reviewers, and disagreements were resolved by discussing and consulting with a third author. The Cochrane risk of bias tool, with reference to random sequence generation, allocation concealment, blinding of participants and personnel, blinding of outcome assessment, incomplete outcome data, selective reporting, and another bias was used to assess the bias of included randomized controlled trials [16]. Each item was rated as low risk of bias, high risk of bias, and unclear risk of bias.

### Quality of evidence

We used the Grading of Recommendations Assessment, Development, and Evaluation (GRADE) guidelines for assessing the strength of evidence of each outcome. As we included RCTs in this meta-analysis, we determined whether to downgrade the quality level of the evidence based on GRADE’s five downgraded criteria with reference to risk of bias, inconsistency, indirectness, imprecision, and publication bias [20]. The result was categorized as high, moderate, low, and very low certainty of evidence.

## Results

### Selection process

**Fig 1:**
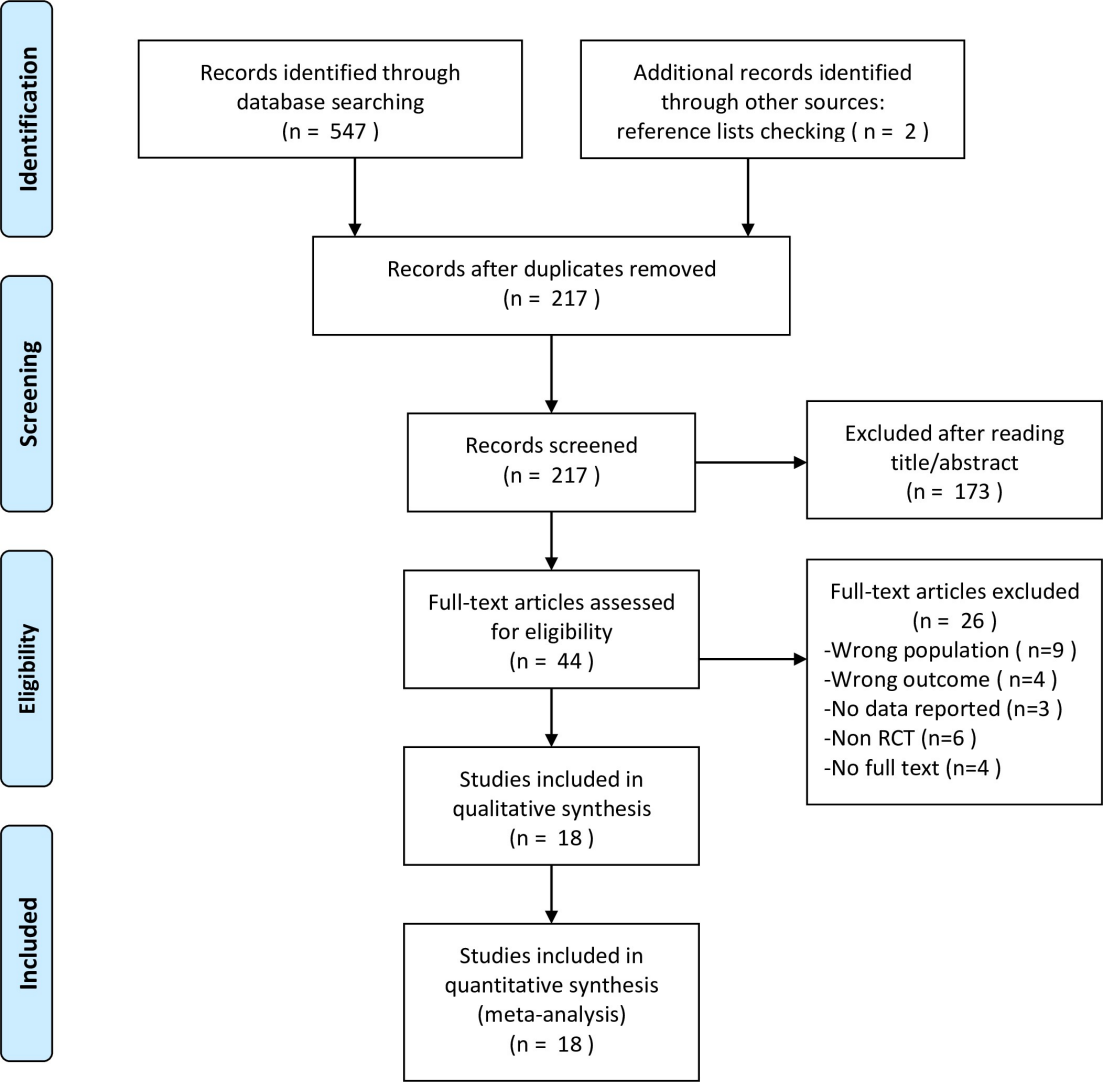


Following the search strategy, the initial search identified 549 articles. After removing duplicated articles, 217 articles were left and then were excluded 173 articles with a Kappa score of 0.82 (95% CI 0.74–0.90) by reading the title and abstract carefully. Next, 26 articles were excluded with an inter-rater agreement Kappa score of 0.91 (95% CI 0.79–1.00) after reading the full text on account of the wrong population (N = 9), not related outcomes (N = 4), no data reported for analysis (N = 3), not RCTs (N = 6), no full text (N = 4). The detailed information about the Kappa score between the reviewers was shown in S1 Table. Consequently, 18 RCTs were included for qualitative and quantitative analysis. The detailed information of the flowchart about searching and including studies was shown in Fig 1.

### Study characteristics

Characteristics of the 18 included randomized controlled trials ranging from 2004 to 2019 were shown in Table 1. Intervention duration ranged from 7 days to 24 weeks. Among these studies, 13 randomized controlled trials [4, 13, 21–31] compared yoga to non-exercise control (e.g. usual care, education), and 8 randomized controlled trials [4, 10–13, 26, 32, 33] compared yoga to traditional physical therapy or exercises. Additionally, 3 trials [4, 13, 26] both compared yoga to non-exercise control and exercises.

**Table 1:** Characteristics of included studies.

Reference, year	Country	Disease diagnosis	Participant characteristics	Intervention duration	Outcome measures	Adverse events
Cox et al., [21] 2010	Britain	CLBP >18m	Yoga group(n = 10): 39 y; 8 females; usual care(n = 10): 51 y; 5 females;	Iyengar yoga: 12 weekly 75-min classes	Pain (Aberdeen Back Pain Scale); Disability (RMDQ); Quality of life (SF-12);	Not reported
Galantino et al., [22] 2004	America	CLBP >6m	Yoga group(n = 11): 30–65 y; No treatment(n = 11): 30–65 y; Males:5; Females:17;	Hatha yoga; 6 weeks, twice per week	Disability (ODI);	Not reported
Groessl et al., [31] 2017	America	CLBP >6m	Yoga group(n = 75): 53.3±12.7 y; 20 females; Usual care(n = 75): 53.6±13.9 y; 19 females;	Hatha yoga; 12 weekly 60-min classes, twice a week	Pain (Brief Pain Inventory); Disability (RMDQ);	Yes
Jacobs et al., [24] 2004	America	CLBP >12m	Yoga group(n = 28): 43.5 y; No reported gender; wait-list(n = 24): 43.2 y: No reported gender;	Iyengar yoga: 12 weekly 90-min classes, twice a week	Pain (VAS); Disability (RMDQ and ODI); Quality of life (SF-36);	Not reported
Kuvacic et al., [23] 2018	Croatia	CLBP >12w	Yoga group(n = 15): 33.6±4.30 y; 6 females; Education(n = 15): 34.7±4.87 y; 8 females;	Yoga: 8 weekly 75-min classes, twice a week	Pain (NPRS); Disability (ODI);	Not reported
Nambi et al., [10] 2014	India	NCLBP >3m	Yoga group(n = 30): 44.26±9.26 y; 19 females; Exercise group(n = 30): 43.66±8.82 y; 13 females;	Iyengar yoga: 4 weeks, 1 h per week at the class and 30 mins, 5 days a week at home	Pain (VAS);	Not reported
Neyaz et al., [12] 2019	India	NCLBP >12w	Yoga group(n = 35): 18–55 y; 17 females; conventional therapeutic exercise(n = 35): 18–55 y; 18 females;	Hatha yoga: 6 weeks, 35-min weekly Hatha yoga sessions	Pain (NPRS); Disability (RMDQ);	Yes
Saper et al., [25] 2009	America	CLBP >12w	Yoga group(n = 15): 44 y; 12 females Usual care group(n = 15): 44 y; 13 females;	Hatha yoga: 12 weekly 75-min classes	Pain (VAS); Disability (RMDQ); Quality of life (SF-36);	Yes
Saper et al., [4] 2017	America	CLBP >12w	Yoga group(n = 127): 46.4±10.4 y; 72 females; Physical therapy(n = 129): 46.4±11 y; 90 females; Education(n = 129): 44.2±10.8 y; 42 females;	Yoga: 12 weekly 75-min classes	Pain (VAS); Disability (RMDQ); Quality of life (SF-36);	Yes
Sherman et al., [13] 2011	America	CLBP >3m	Yoga group(n = 92): 46.6±9.8 y; 62 females; Stretching group(n = 91): 49±9.9 y; 57 females; Self-care group(n = 91): 50.8±9.1 y; 27females;	Viniyoga; 12 weekly 75-min classes	Pain (bothersomeness of pain 0–10 point); Disability (RMDQ);	Yes
Sherman et al., [26] 2005	America	CLBP >3m	Yoga group(n = 36): 44±12 y; 25 females; Exercise(n = 35): 42±15 y; 23 females; Self-care(n = 30): 45±11 y; 21 females;	Viniyoga; 12 weekly 75-min classes	Pain (bothersomeness of pain 0–10 point); Disability (RMDQ);	Not reported
Tekur et al., [11] 2012	India	CLBP >3m	Yoga group(n = 40): 49±3.6 y; 21 females; Physical therapy exercises(n = 40): 48±4 y; 15 females;	Yoga and related practices; 8 h/day for 7 days	Pain (VAS);	Not reported
Tekur et al., [32] 2008	India	CLBP >3m	Yoga group(n = 40): 49±3.6 y; 21 females; Physical exercises(n = 40): 48±4 y; 15 females;	Hatha yoga and related practices; 8 h/day for 7 days	Pain (Oswestry back pain index); Disability (ODI);	Not reported
Teut et al., [27] 2016	India	CLBP >6m	Yoga group(n = 61): 73±5.6 y; 54 females; No treatment(n = 57): 72.6±6 y; 52 females;	Viniyoga: 12 weekly 45-min classes	Pain (VAS); Quality of life (SF-36);	Not reported
Tilbrook et al., [28] 2011	Britain	CLBP >18m	Yoga group(n = 156): 46.4±11.3 y; 106 females; Usual care group(n = 157): 46.3±11.5 y; 114 females;	Yoga; 12 weekly 75-min classes	Pain (Aberdeen Back Pain Scale); Disability (RMDQ); Quality of life (SF-12);	Yes
Wattamwar et al., [33] 2013	India	CLBP >3m	Yoga group(n = 12): 20–50 y; No reported gender; Occupational therapy(n = 12): 20–50 y; No reported gender;	Iyengar yoga; 10 weeks, 45–60 min session	Pain (Oswestry back pain index); Disability (ODI);	Not reported
Williams et al., [29] 2009	America	CLBP >3m	Yoga group(n = 43): 48.4±1.86 y; 22 females; Standard medical care(n = 47): 47.6±1.47 y; 37 females;	Iyengar yoga; 24 weekly 90-min classes, twice a week	Pain (VAS); Disability (ODI);	Not reported
Williams et al., [30] 2005	America	CLBP >3m	Yoga group(n = 20): 48.7±10.6 y; 13 females; Education(n = 24): 48.0±1.96 y; 17 females;	Iyengar yoga; 16 weekly 90-min classes	Pain (VAS);	Yes

CLBP, Chronic low back pain; NCLBP, Nonspecific chronic low back pain; y, years; min, minutes; m, months; w, weeks; h, hours; VAS, Visual Analogue Scale; ODI, Oswestry Disability Index; RMDQ, Roland Morris Disability Questionnaire; SF-36, Medical Outcomes Study Questionnaire Short Form 36 Health Survey; NPRS, Numerical Pain Rating Scale; SF-12, Medical Outcomes Study Questionnaire Short Form 12 Health Survey.

### Outcome measures

There were 17 studies [4, 10–13, 21, 23–33] measuring pain intensity with a Visual Analogue Scale (VAS) [4, 10, 11, 24, 25, 27, 29, 30], Numerical Pain Rating Scale (NPRS) [12, 23], 0–10 points bothersomeness of pain [13, 26], Aberdeen Back Pain Scale [21, 28], Oswestry back pain index [32, 33], Brief Pain Inventory [31]. 6 studies [22–24, 29, 32, 33] used Oswestry Disability Index (ODI), 9 studies [4, 12, 13, 21, 24–26, 28, 31] using Roland-Morris Disability Questionnaire (RMDQ) for disability assessment. 4 studies [4, 24, 25, 27] used SF-36 and 2 studies [21, 28] using SF-12 for quality of life measure.

### Risk of bias assessment

**Fig 2:**
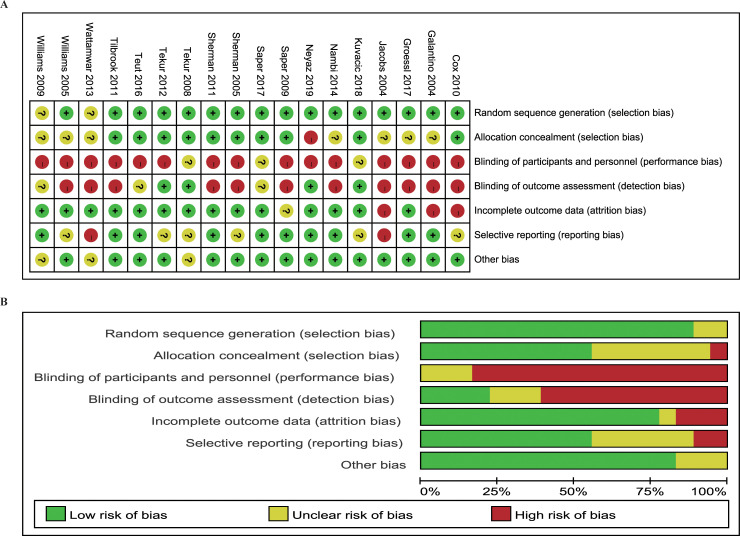


Details of the results about the risk of bias assessment were shown in Fig 2. The main weakness of all included studies was related to the blinding method. There was one study [33] only corresponding to one item of low risk of bias, and sensitivity analysis was performed by excluding this trial to explore the source of heterogeneity.

### Quality of evidence

To our knowledge, it was very difficult to meet in exercise studies where neither the provider, patients nor outcome assessor could be blinded, and these RCTs failed to meet the blinding of participants and outcome assessment. Consequently, the quality of the evidence was downgraded one level due to the RCTs design. Ultimately, the quality of evidence for each outcome was rated as from very low to moderate. The detailed information of the GRADE assessment was shown in the S2 Table.

### Effect on pain relief

#### Yoga versus non-exercise

**Fig 3:**
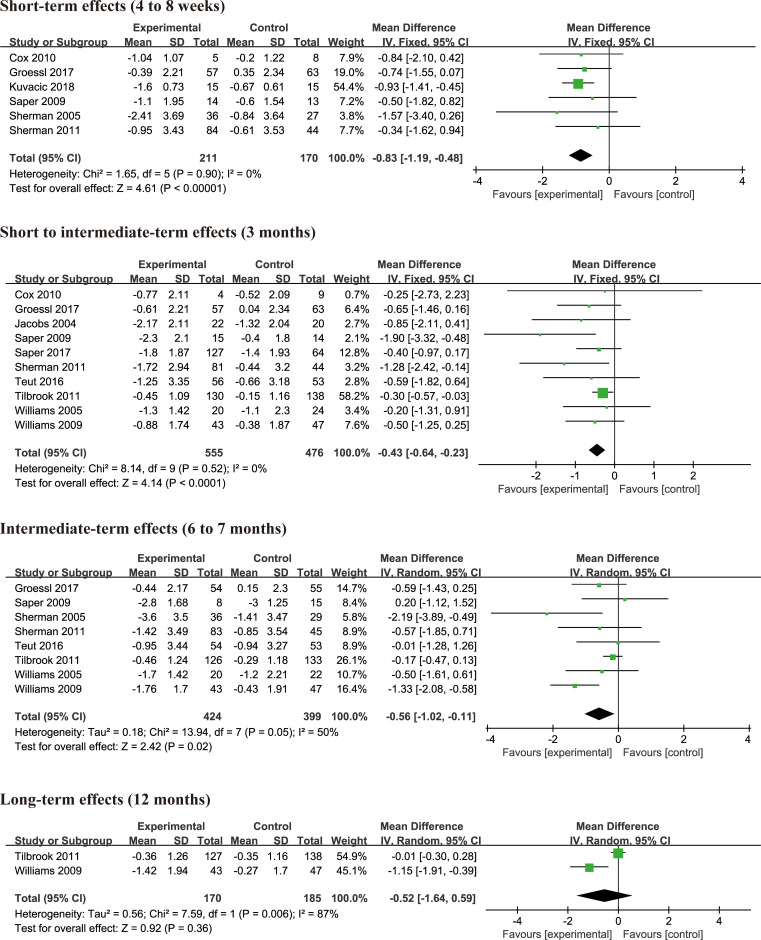


12 studies [4, 13, 21, 23–31] investigated the effects of yoga on pain intensity compared with the non-exercise group (e.g. usual care, education). A total of 2590 participants was involved in the 12 studies that 1360 participants were in the experimental group, and 1230 participants in the control group. The assessment tool for pain was different, including VAS 0 to 10 scale or NPRS 0 to 10 scale, VAS 0 to 100 scale, 0–10 points bothersomeness of pain. Thus, we converted all these scales to a 0 to 10 points scale, and the mean difference with 95% confidence intervals was used to estimate the pooled effects. It suggested that yoga could significantly reduce pain at 4 to 8 weeks (MD = -0.83, 95% CI = -1.19 to -0.48, p<0.00001, I^2^ = 0%), 3 months (MD = -0.43, 95% CI = -0.64 to -0.23, p<0.0001, I^2^ = 0%), 6 to 7 months (MD = -0.56, 95% CI = -1.02 to -0.11, p = 0.02, I^2^ = 50%) compared with non-exercise control (Fig 3). There was no statistically significant difference in pain between two groups at 12 months (MD = -0.52, 95% CI = -1.64 to 0.59, p = 0.36, I^2^ = 87%) (Fig 3). Only two studies [28, 29] examined the effect of yoga compared with non-exercise control on pain in long term (12 months).

#### Yoga versus physical therapy exercise

**Fig 4:**
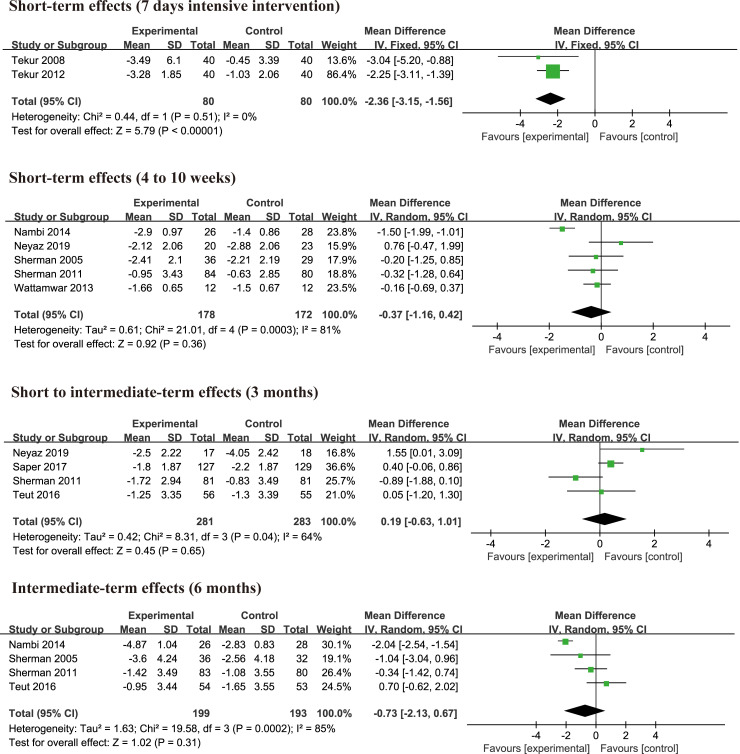


There were 9 studies [4, 10–13, 26, 27, 32, 33] examining the effects of yoga practices on pain compared with physical therapy exercise. A total of 1466 participants was involved in the 9 studies that 738 participants were in the yoga group, and 728 participants in the exercise or physical therapy group. After 7 days of intensive yoga intervention, yoga could significantly reduce pain (MD = -2.36, 95% CI = -3.15 to -1.56, p<0.00001, I^2^ = 0%) (Fig 4) compared with physical therapy exercise. However, there was no significant difference on pain relief at 4 to 10 weeks (MD = -0.37, 95% CI = -1.16 to 0.42, p = 0.36, I^2^ = 81%), 3 months (MD = 0.19 95% CI = -0.63 to 1.01, p = 0.65, I^2^ = 64%), 6 months (MD = -0.73, 95% CI = -2.13 to 0.67, p = 0.31, I^2^ = 85%) (Fig 4) between yoga and control group. Heterogeneity was high, and sensitivity analysis was carried out by removing trials one by one. There was still no statistical difference after sensitivity analysis at 4 to 10 weeks (MD = -0.10, 95% CI = -0.50 to 0.30, p = 0.64, I^2^ = 0%), 3 months (MD = 0.48, 95% CI = -0.08 to 1.03, p = 0.09, I^2^ = 17%), 6 months (MD = -0.10, 95% CI = -0.97 to 0.78, p = 0.83, I^2^ = 18%). Results did not change when studies were excluded one by one from analyses. There were no studies investigating pain in long term. The detailed information was shown in Fig 4.

### Effect on disability changes

#### Yoga versus non-exercise

**Fig 5:**
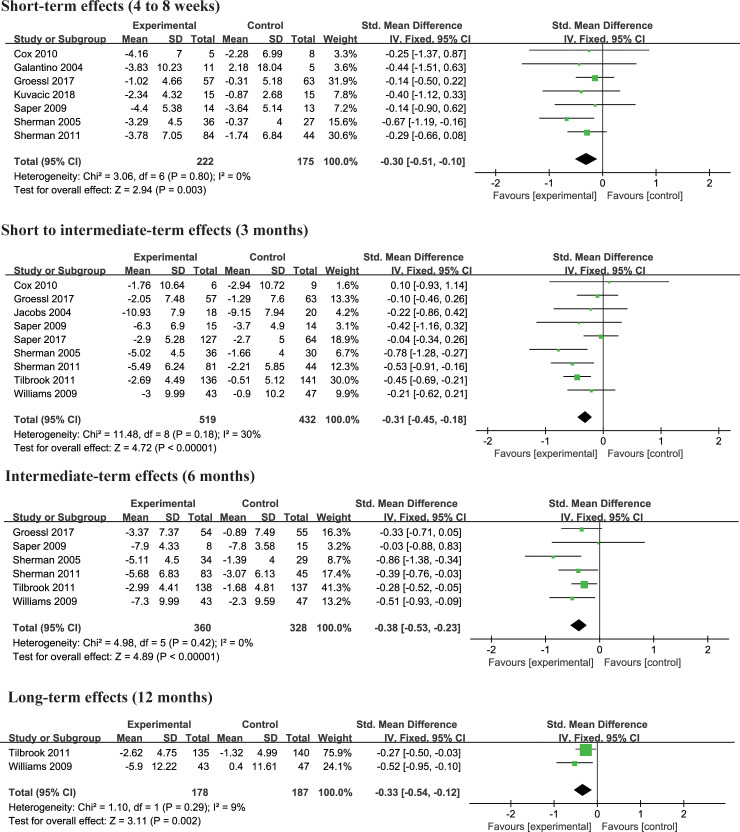


There were 11 randomized controlled trials [4, 13, 21–26, 28, 29, 31] investigating effects of yoga on disability changes compared with non-exercise group by different assessment tools, 3 trials [22, 23, 29] using ODI, 7 trials [4, 13, 21, 25, 26, 28, 31] using RMDQ, one trial [24] using both ODI and RMDQ. Firstly, we performed a meta-analysis with standardized mean difference by pooling ODI and RMDQ simultaneously. Next, subgroup analysis based on Oswestry Disability Index (ODI), Roland Morris Disability Questionnaire (RMDQ) scales respectively, with the mean difference were conducted. The results showed that yoga was better than non-exercise at 4 to 8 weeks (SMD = -0.30, 95% CI = -0.51 to -0.10, p = 0.003, I^2^ = 0%), 3 months (SMD = -0.31, 95% CI = -0.45 to -0.18, p<0.00001, I^2^ = 30%), 6 months (SMD = -0.38, 95% CI = -0.53 to -0.23, p<0.00001, I^2^ = 0%), 12 months (SMD = -0.33, 95% CI = -0.54 to -0.12, p = 0.002, I^2^ = 9%) (Fig 5). For subgroup analysis of RMDQ, yoga could significantly improve disability at 4 to 6 weeks (MD = -1.64, 95% CI = -2.77 to -0.51, p = 0.004, I^2^ = 0%), 3 months (MD = -1.88, 95% CI = -2.60 to -1.17, p<0.00001, I^2^ = 26%), 6 months (MD = -1.88, 95% CI = -2.71 to -1.06, p<0.00001, I^2^ = 28%), 12 months (MD = -1.30, 95% CI = -2.45 to -0.15, p = 0.03) compared with non-exercise (S1 Fig). However, subgroup analysis of ODI, there was no significant difference at 6 to 8 weeks (MD = -1.57, 95% CI = -4.12 to 0.97, p = 0.23, I^2^ = 0%) and 3 months (MD = -1.97, 95% CI = -5.19 to 1.25, p = 0.23, I^2^ = 0%) between two groups (S2 Fig). One study [29] showed that yoga could significantly improve disability on ODI at 6 months (MD = -5.00, 95% CI = -9.05 to -0.95, p = 0.02) and 12 months (MD = -6.30, 95% CI = -11.24 to -1.36, p = 0.01) (S2 Fig). The pooled results did not change when performing sensitivity analysis.

#### Yoga versus physical therapy exercise

**Fig 6:**
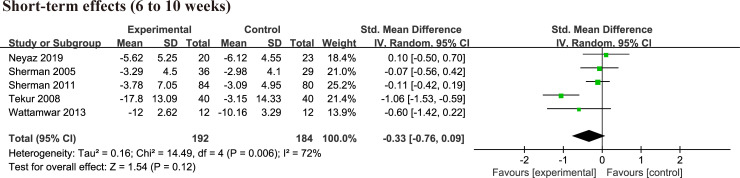


There were 6 randomized controlled trials [4, 12, 13, 26, 32, 33] investigating effects of yoga on disability changes compared with physical therapy exercise group by different assessment tools, 2 trials [32, 33] using ODI, 4 trials [4, 12, 13, 26] using RMDQ. There was no significant difference in RMDQ at 6 week (MD = -0.34, 95% CI = -1.60 to 0.92, p = 0.60, I^2^ = 0%), 3 months (MD = -0.04, 95% CI = -1.76 to 1.67, p = 0.96, I^2^ = 67%), 6months (MD = -1.32, 95% CI = -2.78 to 0.13, p = 0.07, I^2^ = 0%) between yoga and exercise group (S3 Fig). Two studies [32, 33] investigated the short-term effects using ODI, and the pooled result was no significant difference (MD = -7.93, 95% CI = -20.47 to 4.60, p = 0.21, I^2^ = 93%) (S4 Fig). There was no significant difference (SMD = -0.33, 95% CI = -0.76 to 0.09, p = 0.12, I^2^ = 72%) between yoga and exercise group at short term by merging RMDQ and ODI outcome measure (Fig 6). After sensitivity analysis, results did not change (SMD = -0.11, 95% CI = -0.34 to 0.12, p = 0.35, I^2^ = 0%).

### Effect on quality of life

#### Yoga versus non-exercise

**Fig 7:**
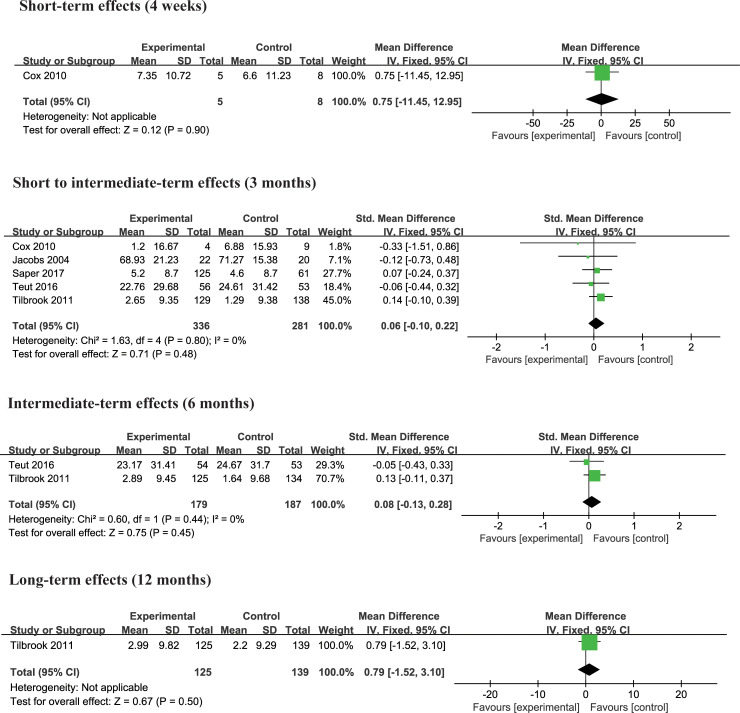


**Fig 8:**
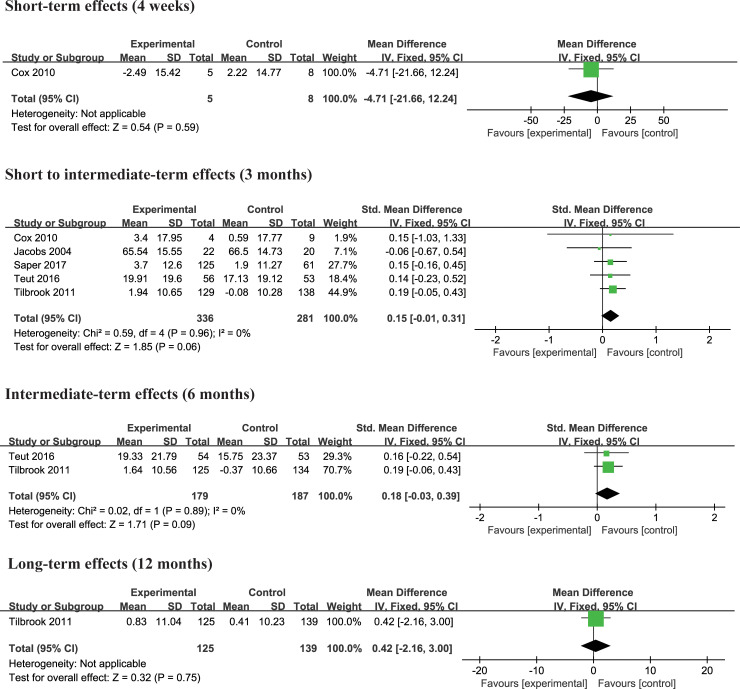


Six trials [4, 21, 24, 25, 27, 28] compared the effects of yoga and non-exercise interventions on physical and mental functioning. When combining the 36 items short form health survey (SF-36) and the 12 items short form health survey (SF-12), the standardized mean difference with 95% confidence intervals was calculated. Besides, we still used the mean difference for pooling effects. The result suggested that yoga had the same effect on the physical quality of life and mental quality of life at different time points as non-exercise (Figs 7 and 8).

#### Yoga versus physical therapy

**Fig 9:**
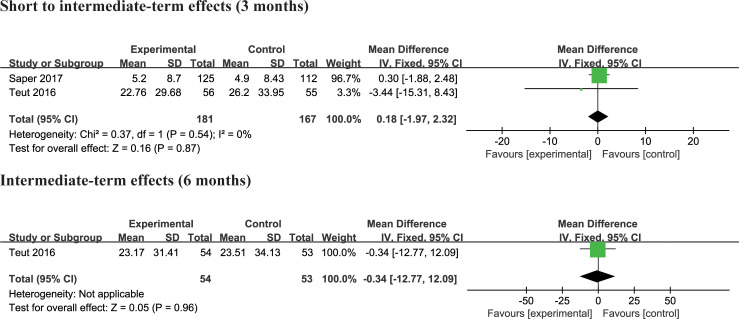


**Fig 10:**
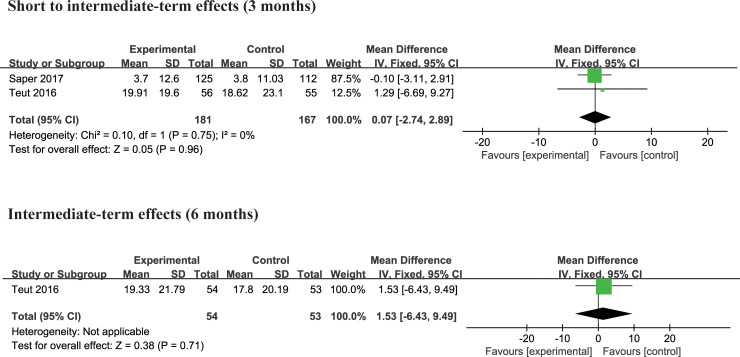


Two trials [4, 27] compared the effects of yoga and physical therapy exercise interventions on physical and mental functioning. There was no significant difference in physical quality of life and mental quality of life at 3 months and 6 months compared to yoga with physical therapy exercise (Figs 9 and 10). No studies reported the effect of yoga at other follow-up periods.

### Publication bias

At least nine studies were included, and the funnel plot was performed to assess the potential publication bias. The funnel plot for pain and disability at 3 months compared yoga with non-exercise control was performed, showing an unclear but rather low risk of publication bias (S5 Fig).

### Adverse events

Seven studies [4, 12, 13, 25, 28, 30, 31] reported adverse events, which patients experienced mild increased back pain in the yoga group, mild or moderate increased low back pain after doing recommended exercises.

## Discussion

Chronic low back pain has a negative effect on millions of people worldwide [34]. Many treatment options exist for CLBP [35], but most have limited effectiveness [36]. Thus, this systematic review and meta-analysis were conducted to evaluate the effects of yoga for CLBP and verify whether was a complementary and alternative treatment for patients with CLBP. We divided the included studies into non-exercise control and physical therapy exercise control. This approach could potentially answer the clinical question about whether CLBP patients should have more exercise-directed treatment like yoga. We defined that outcomes were assessed at short-term (closest to six weeks), short to intermediate-term (closest to three months), intermediate-term (closest to six months), and long-term (closest to one year) follow up periods. This definition could precisely investigate the effects of yoga on CLBP patients at different times and reduce heterogeneous effects by mixing short and long term.

Based on this meta-analysis, we could get moderate evidence that yoga was able to relieve pain at short, short to intermediate, and intermediate term. The result of this review and meta-analysis was consistent with the previous conclusion that yoga could relieve pain and improve disability better than non-exercise, such as no treatment, waiting-list, a minimal intervention (e.g. education, booklets) and usual care. We thought this result was credible just as most studies related effects of yoga on CLBP have shown. However, we found very low-certainty evidence that yoga could not relieve pain at 12 months. This was low-certainty evidence, as only two studies examined the effect of yoga compared with non-exercise control on pain at long term (12 months), and the heterogeneity was high. More studies will need to be conducted in the future to explore the long-term effects of yoga. Compared with physical therapy exercise, there was no significant difference. In other words, it could potentially answer the clinical question that CLBP patients should have more exercise-directed treatment like yoga. Potential mechanisms for these changes include improved flexibility, mobility and stability in muscles and joints, spinal alignment and posture derived from practicing the posture control of yoga, increased mental and physical relaxation derived from practicing meditation and breath control, and improved body awareness gained through both the physical and mental aspects of yoga [30, 37, 38]. It was worth mentioning that Tekur concluded 7 days of an intensive yoga intervention reduced pain and disability in patients with CLBP better than a physical exercise regimen [11, 32]. We did not know why resulted in this. Yoga has generated plenty of interest and attention among the public, and the public health community recommended it as an alternative treatment for some chronic health conditions [15, 39]. Compared to the previous meta-analysis, we included more relevant high-quality RCTs, and the funnel plot for pain and disability at 3 months compared to non-exercise control was performed. It revealed an unclear but rather low risk of publication bias.

For back-specific disability as an outcome, Roland Morris Disability Questionnaire (RMDQ) and Oswestry Disability Index (ODI) often were used for evaluation. Firstly, we performed a meta-analysis with a standardized mean difference by pooling ODI and RMDQ simultaneously. Next, subgroup analysis based on Oswestry Disability Index (ODI), Roland Morris Disability Questionnaire (RMDQ) scales respectively, with the mean difference were conducted. For subgroup analysis of RMDQ, yoga could significantly improve disability at different time points. However, subgroup analysis of ODI, there was no significant difference at 6 to 8 weeks between yoga and non-exercise. There was a debate about it, and yoga indeed could improve disability through using standardized mean difference method for pooling RMDQ and ODI simultaneously. In previous studies, they were uncertain whether yoga offered any advantages over traditional exercise programs in disability. In this meta-analysis, the problem was solved, and yoga played the same role in improving back-related disability as physical therapy exercise programs from short to intermediate term. Unfortunately, no studies investigated the long-term effects of yoga compared with physical therapy exercise.

Interestingly, it was shown that there was a non-significant difference in physical or mental quality of life in individuals with chronic low back pain compared with non-exercise or physical therapy exercise. This important information was not mentioned in previous studies. Many people had different views for this as absent of enough primary studies for pooling. We have tried our best to search as many relevant studies as possible, including unpublished data. We originally searched 7 RCTs [4, 21, 24–28] which used SF-36 or SF-12 to assess the efficacy of yoga in quality of life for CLBP patients, but, two trials [25, 26] only showed no differences in SF-36 scores without providing any data. To solve this doubt, we searched for relevant literature. We found many assessment tools divided into general and specific assessments that were used to assess the treatment effect for chronic low back pain [40]. Cerrada [41] stated that the SF-36 contained multiple response choices for each question and each response choice also included clear descriptions. However, the SF- 36 was not mainly designed for the evaluation of chronic low back pain on account of its poor specificity among CLBP populations [42–44]. Many studies did not only use the SF-36 scale but combined with other specific evaluations including ODI, RMDQ because it did not fully reflect the degree of low back pain [42–44]. Thus, the SF-36 surely had poor reliability of responses to the personal-related quality of life for CLBP patients [41]. For that reason, we had a concern about whether it was appropriate to use SF-36 as a quality of life assessment for patients with low back pain. But, as many RCTs have used SF-36 as a quality of life measure for CLBP patients, we had to include it in the first place as one outcome of this meta-analysis. Here, we emphasized several key findings from this meta-analysis was based on some RCTs using SF-36 for assessing the quality of life. In the future, we expect that more studies could use other scales to assess the quality of life among patients with chronic low back pain.

This meta-analysis also had a few limitations which should be stated. Firstly, although all included studies were RCTs, it was very difficult to meet in exercise studies where neither the provider, patients nor outcome assessor could be blinded, and these RCTs failed to meet the blinding of participants and outcome assessment. Secondly, we did not analyze other cofounding variables, such as the yoga type, the yoga dose and frequency, age, gender. Next, in populations with mild CLBP worldwide, yoga has few side effects in pain relief and improving functional activities [45]. However, seven studies [4, 12, 13, 25, 28, 30, 31] have reported adverse events, and we need to consider its safety and whether has wide applicable human populations. Unfortunately, we did not perform a meta-analysis for this.

## Conclusion

This meta-analysis provided evidence from very low to moderate to investigate the effectiveness of yoga for chronic low back pain patients at different time points. Yoga might decrease pain from short term to intermediate term and improve functional disability status from short term to long term compared with non-exercise (e.g. usual care, education). Yoga had the same effect on pain and disability as any other exercise or physical therapy. Yoga might not improve the physical and mental quality of life based on the result of a merging.

## Supporting information

S1 Checklist PRISMA checklist. (DOC)

S1 Table The Kappa score for selecting studies between the reviewers. (DOC)

S2 Table GRADE Assessment. (DOCX)

S1 Fig Forest plot of yoga versus non-exercise on RMDQ for disability subgroup analysis. (EPS)

S2 Fig Forest plot of yoga versus non-exercise on ODI for disability subgroup analysis. (EPS)

S3 Fig Forest plot of yoga versus physical therapy exercise on RMDQ for disability subgroup analysis. (EPS)

S4 Fig Forest plot of yoga versus physical therapy exercise on ODI for disability subgroup analysis. (EPS)

S5 Fig The funnel plot for pain (A) and disability (B) at 3 months compared yoga with non-exercise.
(EPS)

S1 File The full search strategy of PubMed. (DOC)

S2 File PROSPERO protocol. (PDF)
