# Supplementary material for: Yoga compared to non-exercise or physical therapy exercise on pain, disability, and quality of life for patients with chronic low back pain: A systematic review and meta-analysis of randomized controlled trials
Source: PLoS One. 2020 Sep 1;15(9):e0238544. doi: 10.1371/journal.pone.0238544 (PMC7462307; doi:10.1371/journal.pone.0238544)
Supplement: S1 Table — (DOC) [file pone.0238544.s002.doc]

**S2 Table. The Kappa score for selecting studies between the reviewers.**

**Excluding studies by reading the title and abstract**

| Reviewer 1 |  | Reviewer 2 |  | Total |
| --- | --- | --- | --- | --- |
|  | Exclude | Include | Unclear |  |
| Exclude | 156 | 5 | 3 | 164 |
| Include | 1 | 46 | 2 | 49 |
| Unclear | 3 | 1 | 0 | 4 |
| Total | 160 | 52 | 5 | 217 |

Kappa score: 0.82 (0.74-0.90)

**Excluding studies by reading the full text**

| Reviewer 1 |  | Reviewer 2 |  | Total |
| --- | --- | --- | --- | --- |
|  | Exclude | Include | Unclear |  |
| Exclude | 24 | 0 | 0 | 24 |
| Include | 1 | 18 | 0 | 19 |
| Unclear | 1 | 0 | 0 | 1 |
| Total | 26 | 18 | 0 | 44 |

Kappa score: 0.91 (0.79-1.00)
