# Supplementary material for: Yoga compared to non-exercise or physical therapy exercise on pain, disability, and quality of life for patients with chronic low back pain: A systematic review and meta-analysis of randomized controlled trials
Source: PLoS One. 2020 Sep 1;15(9):e0238544. doi: 10.1371/journal.pone.0238544 (PMC7462307; doi:10.1371/journal.pone.0238544)
Supplement: S2 Table — (DOCX) [file pone.0238544.s003.docx]

**S3 Table. GRADE Assessment****.**

**Yoga versus non-exercise**

| Outcomes | Study  Design | Risk of Bias | Inconsistency | Indirectness | Imprecision | Other  Considerations | No. of Participants | | Absolute Effect (95% CI) | Quality |
| --- | --- | --- | --- | --- | --- | --- | --- | --- | --- | --- |
|  | | | | | | | Yoga | Control |  |  |
| Pain at short term | RCTs | Serious | No | No | No | No | 211 | 170 | MD -0.83 (-1.19 to -0.48) | Moderate |
| Pain at short to intermediate term | RCTs | Serious | No | No | No | No | 555 | 476 | MD -0.43 (-0.64 to -0.23) | Moderate |
| Pain at intermediate term | RCTs | Serious | No | No | Serious | No | 424 | 399 | MD -0.56 (-1.02 to -0.11) | Low |
| Pain at long term | RCTs | Serious | No | No | Serious | Serious | 170 | 185 | MD -0.52 (-1.64 to 0.59) | Very low |
| Disability at short term | RCTs | Serious | No | No | No | No | 222 | 175 | SMD -0.30 (-0.51 to -0.10) | Moderate |
| Disability at short to intermediate term | RCTs | Serious | No | No | No | No | 519 | 432 | SMD -0.31 (-0.45 to -0.18) | Moderate |
| Disability at intermediate term | RCTs | Serious | No | No | No | No | 360 | 328 | SMD -0.38 (-0.53 to -0.23) | Moderate |
| Disability at long term | RCTs | Serious | No | No | No | Serious | 178 | 187 | SMD -0.33 (-0.54 to -0.12) | Low |
| Physical quality of life at short term | RCTs | Serious | Serious | No | No | Serious | 5 | 8 | MD 0.75 (-11.45 to 12.95) | Very low |
| Physical quality of life at short to intermediate term | RCTs | Serious | No | No | No | No | 336 | 281 | SMD 0.06 (-0.10 to -0.22) | Moderate |
| Physical quality of life at intermediate term | RCTs | Serious | No | No | No | No | 179 | 187 | SMD 0.08 (-0.13 to 0.28) | Moderate |
| Physical quality of life at long term | RCTs | Serious | No | No | No | Serious | 125 | 139 | MD 0.79 (-1.52 to 3.10) | Low |
| Mental quality of life at short term | RCTs | Serious | Serious | No | No | Serious | 5 | 8 | MD -4.71 (-21.66 to 12.24) | Very low |
| Mental quality of life at short to intermediate term | RCTs | Serious | No | No | No | No | 336 | 281 | SMD 0.15 (-0.01 to 0.31) | Moderate |
| Mental quality of life at intermediate term | RCTs | Serious | No | No | No | No | 179 | 187 | SMD 0.18 (-0.03 to -0.39) | Moderate |
| Mental quality of life at long term | RCTs | Serious | No | No | No | Serious | 125 | 139 | MD 0.42 (-2.16 to 3.00) | Low |

**Yoga versus physical therapy exercise**

| Outcomes | Study  Design | Risk of Bias | Inconsistency | Indirectness | Imprecision | Other  Considerations | No. of Participants | | Absolute Effect (95% CI) | Quality |
| --- | --- | --- | --- | --- | --- | --- | --- | --- | --- | --- |
|  | | | | | | | Yoga | Control |  |  |
| Pain at 7 days intensive intervention | RCTs | Serious | No | No | No | Serious | 80 | 80 | MD -2.36 (-3.15 to -1.56) | Low |
| Pain at short term | RCTs | Serious | No | No | Serious | No | 178 | 172 | MD -0.37 (-1.16 to 0.42) | Low |
| Pain at short to intermediate term | RCTs | Serious | No | No | No | No | 281 | 283 | MD 0.19 (-0.63 to 1.01) | Moderate |
| Pain at intermediate term | RCTs | Serious | No | No | Serious | No | 199 | 193 | MD -0.73 (-2.13 to 0.67) | Low |
| Disability at short term | RCTs | Serious | No | No | No | No | 192 | 184 | SMD -0.33 (-0.76 to -0.09) | Moderate |
| Disability at short to intermediate term | RCTs | Serious | No | No | No | No | 261 | 258 | MD -0.04 (-1.76 to 1.67) | Moderate |
| Disability at intermediate term | RCTs | Serious | No | No | No | Serious | 117 | 112 | MD -1.32 (-2.78 to 0.13) | Low |
| Physical quality of life at short to intermediate term | RCTs | Serious | No | No | No | No | 181 | 167 | MD 0.18 (-1.97 to 2.32) | Moderate |
| Physical quality of life at intermediate term | RCTs | Serious | No | No | No | Serious | 54 | 53 | MD -0.34 (-12.77 to 12.09) | Low |
| Mental quality of life at short to intermediate term | RCTs | Serious | No | No | No | No | 181 | 167 | MD 0.07 (-2.74 to 2.89) | Moderate |
| Mental quality of life at intermediate term | RCTs | Serious | No | No | No | Serious | 54 | 53 | MD 1.53 (-6.43 to -9.49) | Low |

RCTs, randomized controlled trials. MD, mean difference. SMD, standardized mean difference.
