# Supplementary material for: Yoga compared to non-exercise or physical therapy exercise on pain, disability, and quality of life for patients with chronic low back pain: A systematic review and meta-analysis of randomized controlled trials
Source: PLoS One. 2020 Sep 1;15(9):e0238544. doi: 10.1371/journal.pone.0238544 (PMC7462307; doi:10.1371/journal.pone.0238544)
Supplement: S1 File — (DOC) [file pone.0238544.s009.doc]

**S1 File. The full search strategy of PubMed.**

((("Low Back Pain"[Mesh]) OR (low back pain[Title/Abstract] OR low back pains[Title/Abstract] OR lumbago[Title/Abstract] OR lower back pain[Title/Abstract] OR lower back pains[Title/Abstract] OR low back ache[Title/Abstract] OR low back aches[Title/Abstract] OR low backache[Title/Abstract] OR low backaches[Title/Abstract] OR lumbar pain[Title/Abstract] OR herniated disk[Title/Abstract] OR herniated disc[Title/Abstract] OR hernia intervertebral disc[Title/Abstract] OR lumbar degenerat*[Title/Abstract] OR backache[Title/Abstract] OR back disorders[Title/Abstract] OR sciatica[Title/Abstract] OR coccyx[Title/Abstract] OR coccy*[Title/Abstract] OR spondylosis[Title/Abstract])) AND (("Yoga"[Mesh]) OR (yoga[Title/Abstract] OR yogic[Title/Abstract] OR yogi[Title/Abstract] OR yog*[Title/Abstract]))) AND (("Controlled Clinical Trial" [Publication Type] OR "Randomized Controlled Trial" [Publication Type]) OR (randomized controlled trial[Title/Abstract] OR controlled clinical trial[Title/Abstract] OR random allocation[Title/Abstract] OR random*[Title/Abstract] OR placebo[Title/Abstract]))
